# Polyethylene Polyamine-Modified Chitosan Aerogels: Enhanced CO_2_ Adsorbents with Lamellar Porous Structures

**DOI:** 10.3390/polym17030414

**Published:** 2025-02-04

**Authors:** Hui Ming, Haoxin Jiang, Ruiyang Zheng, Mei Wu, Hongying Li, Zhengxin Li, Xudong Zhang, Zihao Yuan, Ziyue Wang

**Affiliations:** State Key Laboratory of Heavy Oil Processing, College of Engineering, China University of Petroleum-Beijing at Karamay, Karamay 834000, China; jhx@st.cupk.edu.cn (H.J.); 2022015757@st.cupk.edu.cn (R.Z.); 2016592017@cupk.edu.cn (M.W.); 2022015793@st.cupk.edu.cn (H.L.); 2022015710@st.cupk.edu.cn (Z.L.); zxd15831351328@163.com (X.Z.); 2022015789@st.cupk.edu.cn (Z.Y.); 2022015733@st.cupk.edu.cn (Z.W.)

**Keywords:** laminar porous structure, chitosan aerogel, polyethylene polyamine, carbon dioxide adsorption

## Abstract

Due to the continuous growth of global carbon dioxide emissions, the development of cost-effective carbon dioxide capture technology has attracted extensive attention. Amino-modified chitosan aerogels with lamellar porous structures are good candidates as carbon dioxide adsorbents because of their degradable properties and low energy consumption. Polyethylene polyamine-modified chitosan aerogels (PEPA-CSs) were prepared through a process of crosslinking and freeze-drying using a chitosan solution, polyethylene polyamine (PEPA), and epichlorohydrin (ECH) as raw materials. The amino group of PEPA was proven to be successfully grafted on the chitosan surface by FITR and XPS. The SEM and TEM analysis showed a rich three-dimensional porous structure and a good rigidity and bearing capacity of the PEPA-CS. The adsorption capacity was significantly increased by PEPA grafting with a maximum value of 1.59 mmol/g at 25 °C and 1 bar through both physical and chemical interactions, which indicates a potential for broad application prospects in industrial CO_2_-capture applications.

## 1. Introduction

Since the Industrial Revolution, human activities have significantly increased greenhouse gas emissions, with carbon dioxide (CO_2_) emerging as the primary gas contributing to global warming [1]. This increase has profoundly impacted global climate change. The urgency of carbon dioxide capture for environmental protection has grown due to the rising atmospheric concentrations of CO_2_, largely attributed to human activities such as the burning of fossil fuels. Carbon capture and storage (CCS) stands out as one of the most significant measures for CO_2_ reduction worldwide. A report by the International Energy Agency (IEA) [2] suggests that CCS could potentially reduce emissions by as much as 8 billion tonnes. In recent years, there has been a significant push to develop innovative CCS technologies and processes to enhance their effectiveness in addressing this critical environmental challenge.

The principal methods for capturing CO_2_ before combustion include the solution absorption method, the solid adsorption method, the membrane separation method, and the low-temperature fractionation method [3]. Following combustion, CO_2_ capture processes comprise chemical solution absorption, chemical adsorption, physical adsorption, and membrane separation [4]. Currently, the solution absorption method is predominantly utilized in industrial settings. Despite its benefits, such as a large processing capacity, high selectivity, and well-established technology, this method faces challenges like high energy consumption and susceptibility to equipment corrosion [5]. To surmount the limitations inherent in traditional solvent methods, researchers have been extensively investigating solid adsorbents. Materials like zeolite, activated carbon, and metal–organic frameworks (MOFs) are employed for the physical adsorption of CO_2_ and can address some of the drawbacks associated with solution-based methods. However, these materials tend to have poor selectivity for CO_2_ adsorption in the presence of mixed gases, and water vapor can adversely affect their adsorption capabilities [6,7,8]. Amino-functionalization presents an effective solution to the issue of poor selectivity in porous adsorbents. By introducing amino groups, this approach aims to enhance the materials’ affinity for CO_2_, thereby improving their performance in capturing carbon dioxide from gas mixtures and counteracting the negative influence of water vapor. This strategy is a promising step towards the development of more efficient and selective CO_2_ capture technologies.

As a new type of porous material, amino-modified aerogel has shown great potential in the field of carbon dioxide adsorption due to its excellent pore structure and high specific surface area. Amino-functionalization is an effective means of improving the CO_2_ adsorption capacity of aerogels, and amino-modified aerogels with excellent CO_2_ adsorption properties can be prepared by impregnation, surface grafting, and in situ polymerization [9]. These advanced materials leverage the unique properties of amino groups to selectively capture CO_2_ molecules. The high surface area of the aerogel provides an abundance of active sites for adsorption, while the porous structure facilitates the diffusion of CO_2_ into the material. By incorporating amino groups, the affinity of the aerogel for CO_2_ is significantly increased, leading to improved adsorption performance.

Chitin, or its deacetylated derivative, chitosan, is a naturally occurring biopolymer (second only to cellulose) that can be used in biomedicine, pharmaceutical, cosmetic, food industry, agriculture, and wastewater treatment [10,11]. Chitosan is a linear polysaccharide obtained by removing acetyl functional groups and liberating amino groups from the backbone, which is a value-added functional biomaterial used in biorefining. It is a copolymer formed by the combination of N-acetyl-D-glucosamine and D-glucosamine by β-(1-4)-glycosidic bonds, and the degree of deacetylation (DD%) (i.e., d-glucosamine content) of chitosan should reach at least 60% [10,12]. Meanwhile, chitosan possesses a certain number of amino groups, which gives it an advantage over ordinary porous materials in absorbing carbon dioxide. Grafting amino groups onto chitosan aerogels can further increase the loading rate of amino groups, thereby enhancing their ability to absorb carbon dioxide.

Alhwaige et al. [13] significantly increased the BET surface area from 153 m^2^·g^−1^ to 415 m^2^·g^−1^ by loading 20 wt% GO in a carbonized CS sorbent. After adding 20 wt% GO, the amount of adsorbed CO_2_ increased from 1.92 mol·kg^−1^ to 4.15 mol·kg^−1^ at 25 °C. Hsan et al. [14] demonstrated that the aerogels fabricated through the grafting of graphene oxide onto CS possess a well-ordered mesoporous architecture. The adsorption capacity of CO_2_ gas by CS-grafted graphene oxide aerogel at 1 bar was about 0.257 mmol·g^−1^, which was far better than that of pure CS. Junpei Fujiki et al. [15] demonstrated that the CO_2_ adsorption capacity of polyethylenimine-grafted chitosan (PEI−CS) was 2.3 mmol·g^−1^ under conditions of 313 K and 15 kPa CO_2_, which significantly increased to 3.6 mmol·g^−1^ in the presence of water vapor. Zhiyan Liu et al. [16] prepared a K2SO4/CS aerogel by radiation-initiated polymerization, which efficiently captured CO_2_ 67.9 mg·g^−1^ at 298 K, 0.1 MPa. Song et al. [17] prepared a quaternary ammonium chitosan (QCS)/polyvinyl alcohol (PVA) hybrid aerogel with a CO_2_ capture capacity of approximately 0.18 mmol·g^−1^ at room temperature. Santosh Kumar et al. [18] dispersed GO into the CS matrix in the form of a nanocomposite membrane and observed an adsorption capacity of 1.0152 mmol·g^−1^ at 4.6 bar.

Among amino modifiers, PEPA stands out as being of particular interest. The results have shown that the use of PEPA as a modifier significantly improves the adsorption capacity and selectivity of cellulose aerogels for CO_2_. For instance, a study demonstrated that amino-functionalized nanocellulose aerogels modified with PEPA exhibited excellent CO_2_ adsorption and effective CO_2_/CH_4_ mixed gas separation properties, thereby highlighting the benefits of PEPA in enhancing aerogel adsorption performance [19]. Furthermore, PEPA-modified aerogels not only maintain a high adsorption capacity but also possess good desorption and recycling properties [20]. This enhancement in adsorption performance is attributed not only to the abundant amino functional groups and high reactivity of PEPA but also to its environmental friendliness and excellent cycling stability, which indicates great potential for practical applications. However, there is a dearth of research on the modification of chitosan aerogels with PEPA as an amino modifier. Consequently, this paper focuses primarily on studying the ability of PEPA-modified chitosan aerogels to adsorb carbon dioxide.

## 2. Materials and Methods

### 2.1. Materials

Chitosan (CS > 400 mPa·s), Polyethyene polyamine (PEPA, AR), Acetic acid (AR, 99.5%), Sodium hydroxide (NaOH, AR, 99%, granulated), Epichlorohydrin (ECH, 99%), and Glutaraldehyde (GA, 50 wt% in water) were all purchased from Beijing Innokai Technology Co., Ltd. (Beijing, China). All chemicals are analytical grade and can be used without further purification.

### 2.2. Design and Synthesis Composite Absorbents

#### 2.2.1. Preparation of Epichlorohydrin Cross-Linked PEPA-Grafted Chitosan Aerogels

PEPA-CS aerogels were prepared using a modified method reported by Qian Minjie et al. [21]. A total of 2 g of CS was dissolved in 100 mL of acetic acid solution with a volume fraction of 1.5%, and then a chitosan–acetic acid solution was added dropwise with a syringe to 100 mL of 1 mol·L-1 sodium hydroxide solution to solidify it into a ball. After washing it several times with deionized water and ethanol, it was transferred to a three-mouth flask, and 10 mL of ethanol was added. A total of 5 mL of epichlorohydrin was added to the water bath at 40 °C and mechanical stirring for 24 h, followed by 3, 7, 11, 15, and 19 mL of PEPA, and the water bath temperature was adjusted to 60 °C for another 6 h. After the reaction, it was washed with deionized water until neutral. It was then freeze-dried at −55 °C to form aerogels, which were named PEPA-3, PEPA-7, PEPA-11, PEPA-15, and PEPA-19.

#### 2.2.2. Preparation of Glutaraldehyde Cross-Linked PEPA Grafted Chitosan Aerogels

**Figure 1:**
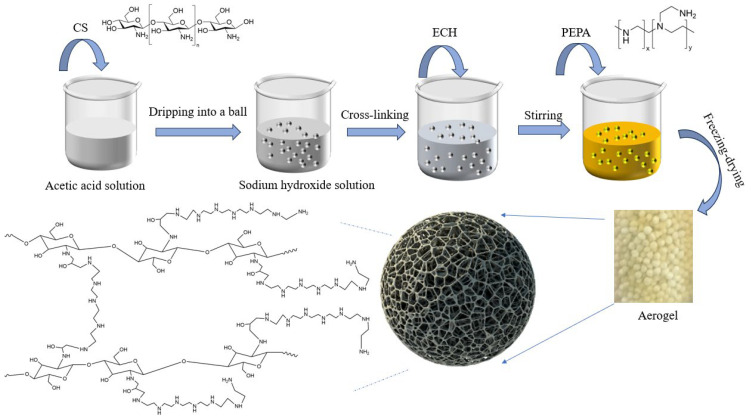
Schematic of the preparation process and structural units of PEPA-CS.

A total of 1.5 g of chitosan (CS) was first dissolved in 100 mL of a 2% (*v*/*v*) acetic acid solution under continuous stirring. Subsequently, PEPA was added to the solution, which was then stirred until complete dissolution was achieved. The resulting clear solution was subjected to ultrasonic degassing in a water bath to remove any residual bubbles. The degassed solution was then carefully introduced into a 20 g/L (NaOH) solution using a syringe at a drop rate of 0.6 mL/min, which facilitated the solidification of the microspheres. Following this, 100 mL of a 2% GA solution was added to the mixture, which was then mechanically stirred in a constant temperature water bath at 50 °C for 4.5 h at a stirring speed of 100 r/min. Finally, the product was washed with deionized water using conventional filtration and subsequently freeze-dried to obtain the desired aerogel. The specific experimental procedures are illustrated in Figure 1. 

### 2.3. Characterization

#### 2.3.1. Fourier Transform Infrared (FTIR) Spectra

The potassium bromide tableting method was used to detect the changes of functional groups in the modified aerogel by Fourier transform infrared (Bruker Vertex 70, Bruker Optics, Billerica, MA, USA), and the successful grafting of the amino modifier was speculated.

#### 2.3.2. Thermogravimetric Analysis (TGA)

TGA was carried out on TA TGA55 (New Castle, DE, USA). The weight loss of the sample was scanned from 30 to 800 °C at a heating rate of 10 °C/min. Raise to 800 °C at 10 mL/min to avoid oxidation of the sample.

#### 2.3.3. Scanning Electron Microscopy (SEM)

SEM was detected by the Zeiss Sigma 300 (Carl Zeiss Microscopy GmbH, Jena, Thuringia, Germany)instrument in Germany. The samples were sprayed with gold, and the image information of aerogels of 1 microns, 3 microns, and 5 microns was obtained on the SEM electron microscope stage, and the microstructure of the aerogels was observed.

#### 2.3.4. Transmission Electron Microscope (TEM)

The samples were carried out on FEI Talos F200x (Thermo Fisher Scientific, Hillsboro, OR, USA). Transmission electron microscopy (TEM) was used to study the structure and size of PEPA-CS.

#### 2.3.5. The Brunauer–Emmett–Teller Specific Surface Area (BET)

The specific surface area of N_2_ adsorption was determined by the BET (Micromeritics ASAP 2460, Micromeritics Instrument Corporation, Norcross, GA, USA) equation. The nitrogen adsorption and desorption isotherm uses a surface area and pore size analyzer (ASAP-2460 analyzer Micromeritics, USA).

#### 2.3.6. X-Ray Photoelectron Spectroscopy (XPS)

In order to investigate the surface of the samples, the XPS analyses were conducted by using a Thermo Scientific K-Alpha spectrometer (Thermo Fisher Scientific Inc, Waltham, MA, USA) that was fitted with a monochromatic small-spot X-ray source and a 180 double-focusing hemispherical analyzer with a 128-channel detector [22].

#### 2.3.7. CO_2_ Adsorption and Desorption Capacity

**Figure 2:**
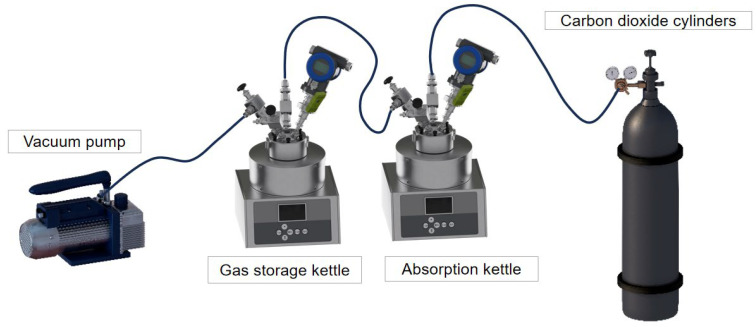
Diagram of carbon dioxide absorption unit.

The product is tested by the carbon dioxide absorption kettle device independently assembled in the laboratory, as depicted in Figure 2, the pressure in the kettle is recorded in real-time through cloud monitoring during the experiment, the carbon dioxide absorption is indirectly tested through the change of pressure or current value, and, finally, the carbon dioxide absorption is calculated through the real gas equation of state.

The calculation is carried out by Equation (1), in which the real gas compression factor is calculated by Peng and Robinson [23], who proposed the PR Equation (2) of state in 1976.

(1) PV=ZnRT

(2) $$p=\frac{RT}{v-b}-\frac{a}{v\left(v+b\right)+b(v-b)}$$

where *R* represents the universal gas constant (8.314472 J·mol^−1^·K^−1^); *T* is the temperature (K); *v* is the molar volume(cm^3^·mol^−1^); *p* is the pressure (MPa).

The two parameters *a* (3) and *b* (4) represent the energy parameter (J·cm^3^·mol^−2^) and covolume (cm^3^·mol^−1^), and their expressions are shown:

(3) $$a=0.45724\frac{R^{2}T_{c}^{2}}{P_{c}}a\left(T\right)$$

(4) $$b=0.0778\frac{RT_{c}}{P_{c}}a(T)$$

(5) $$a\left(T\right)={\left[1+(0.37646+1.54226\omega -0.26992\omega^{2})(1-T_{r}^{0.5})\right]}^{2}$$

where *T_c_* and *P_c_* denote the critical temperature (K), and critical pressure (MPa), respectively. *ω* is the acentric factor and *T_r_ = T/T_c_* is the reduced temperature.

## 3. Results

### 3.1. Effect of Different Variables on Amino Grafting

In this experiment, the effects of chitosan dosage, amino modifier dosage, and crosslinking time on amino grafting were studied.

**Figure 3:**
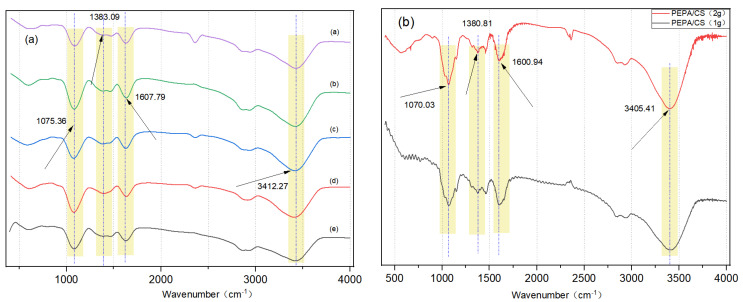

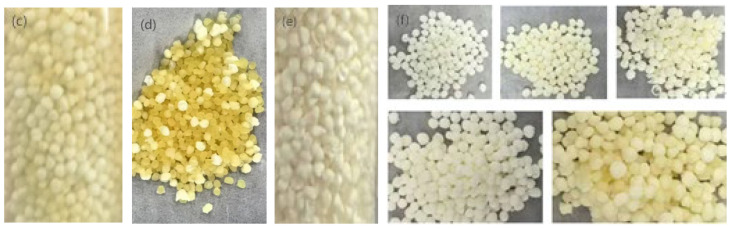
(**a**) FTIR spectra of a. PEPA-19, b. PEPA-15, c. PEPA-11, d. PEPA-7, e. PEPA-3. (**b**) FTIR spectra of modified aerogels with 2 g chitosan cross-linked 24 h and 1 g chitosan cross-linked 24 h. (**c**–**e**) Physical diagram of 2 g chitosan cross-linked 24 h modified aerogel. Physical diagram of 2 g chitosan cross-linked 12 h modified aerogel. Physical diagram of 1 g chitosan cross-linked 24 h modified aerogel. (**f**) Physical diagram of PEPA-3, PEPA-7, PEPA-11, PEPA-15, PEPA-19.

When the chitosan amount was increased from 1 g to 2 g while keeping the modifier concentration constant, there was a significant increase in the peak intensity at 3420 cm^−1^. This increase was attributed to the rise in -NH- groups due to the amino groups present in chitosan itself. During the preparation of chitosan hydrogels using 1 g and 2 g of chitosan as substrates, it was observed that the ball-forming effect was poor, the coagulation time was prolonged, and the process was unsuitable for molding. Concurrently, a 3 g chitosan hydrogel was prepared; however, during the coagulation process, when attempting to form drops through a syringe, the solution’s viscosity was too high, and the dropping rate was too slow. Therefore, 2 g of chitosan was chosen as the substrate for hydrogel preparation. Additionally, according to the literature, GA is the primary crosslinking agent for chitosan, and ECH is also used for crosslinking [24]. However, most of these crosslinking agents are utilized for the adsorption of metal ions. Aerogel pellets prepared with GA were poorly formed, had a yellowish color, and contained more fragments, whereas those prepared with ECH exhibited good ball formation and fewer fragments. Subsequently, the effect of cross-linking time with the cross-linking agent on the preparation of chitosan aerogel was investigated. It was observed that aerogel pellets cross-linked for 12 h were more cake-like and lighter in color, while those cross-linked for 4 h had a better pelleting effect and a darker color. Using 2 g of chitosan and cross-linking for 24 h resulted in the best spheroidal effect. The aerogel pellets from PEPA-3 to PEPA-19 (Figure 3f) progressively darkened, indicating an increasingly better grafting effect. Infrared analysis of these five substances revealed that PEPA-15 had the deepest amino peak, signifying the best grafting effect. According to the physical map analysis, a 2 g chitosan substrate cross-linked for 24 h provided the best spheroidal effect. The color of the aerogel pellets from PEPA-3 to PEPA-19 became progressively darker, which suggests an increasingly better grafting effect. The optimal conditions for the synthesis of aerogels were established as PEPA-15, with a reaction duration of 6 h and a cross-linking period of 24 h. Considering that the water bath temperature is generally lower than the reaction temperature, the reaction temperature was precisely determined to be 65 °C through a meticulous comparison of the actual temperature deviations.

### 3.2. Surface Functional Group Analyses of the Samples

**Figure 4:**
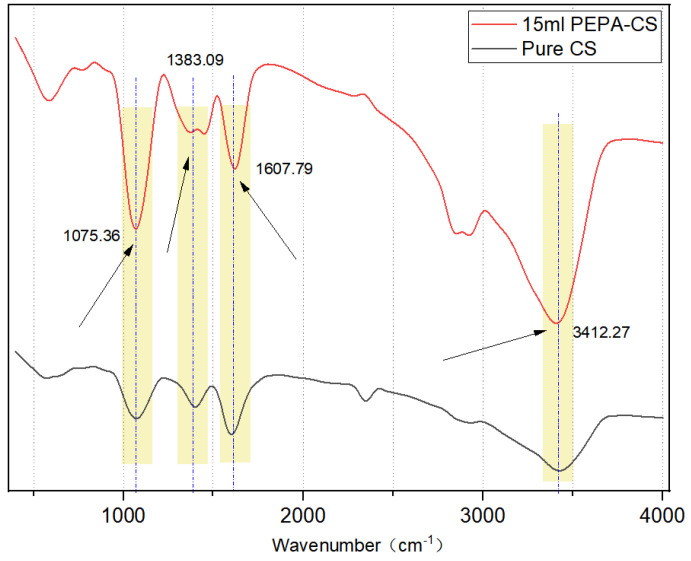
FTIR spectra of PEPA-15 and pure CS aerogel.

The results of FTIR spectroscopy are shown in Figure 4. The wide absorption peak at 3412 cm^−1^ belongs to the expansion vibrations of -OH, -NH_2,_ and -NH-. The peak at 1607 cm^−1^ is attributed to the -C=O- stretching vibration of a small amount of chitin monomer in CS.

The peak at 1414 cm^−1^ can be attributed to the C-N tensile vibration and the peak C-O telescopic vibration at 1075 cm^−1^, 1383 cm^−1^ -COO− sym str and/or O−H def carbamate and/or carbamic acid [25,26]. The peak intensity at 3412 cm^−1^ increased significantly, which was caused by the addition of a large amount of -NH- after PEPA modification of CS, which proved that the amino grafting was successful. The appearance of the band at 1075 cm^−1^, attributed to a C-O stretching vibration, also proved that PEPA was successfully grafted on the CS surface [27].

In this experiment, variable experiments were carried out on the dosage of chitosan in the basement, and the wide absorption peak at 3412 cm^−1^ belonged to the expansion vibration of -OH, -NH_2,_ and -NH-. The peak at 1607 cm^−1^ is attributed to the C=O stretching vibration of a small amount of chitin monomer in CS.

### 3.3. Thermal Gravimetric Analysis of PEPA-CS

The thermal stability of the prepared aerogel is a critical parameter that determines its suitability as a CO_2_ adsorbent. Upon comparing the thermogravimetric curves of pure chitosan aerogels with those of the modified aerogels, it is observed that the latter possesses better thermal stability and a higher decomposition temperature.

**Figure 5:**
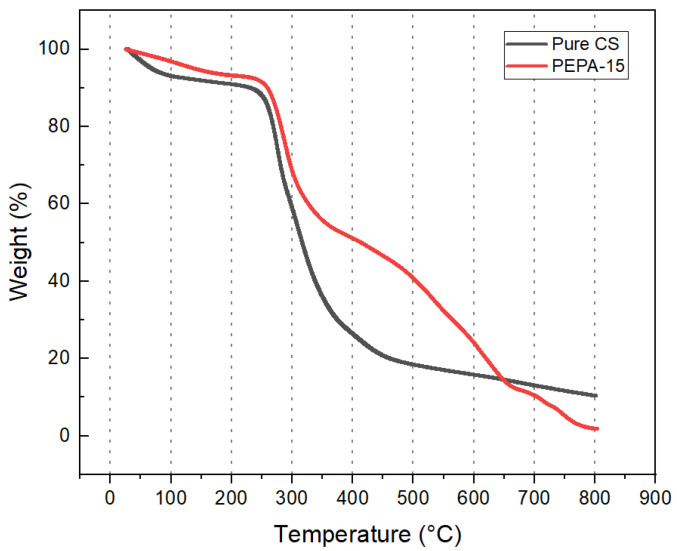
Thermogravimetric curves of PEPA-CS aerogel.

It can be seen from the weight loss curve of PEPA-modified aerogel pellets that the weight loss process can be roughly divided into three stages. The specific procedure of the TG analysis is illustrated in Figure 5. In the first stage, from room temperature to 276 °C, only a slight mass loss of about 8.9% was observed in CS, mainly due to the volatilization of bound water, adsorbed water, and physisorpted gases from the sample. The second stage has a mass loss of about 30% from 276 °C to 800 °C, with the highest rate of mass loss around 311 °C. The weight loss at this stage is attributed to the decomposition of heteroatoms in CS. In the third stage, the product mainly corresponds to the loss of 44.9% of the grafted PEPA and crosslinker ECH. The product begins to decompose at about 276 °C CS, which is easy to desorb and reuse compared with more aerogel products, with a higher stable temperature [21]. The amine grafting efficiency of the experimentally prepared amine-modified aerogel, as determined by the gravimetric method, was found to be 60.5%.

### 3.4. Morphology of PEPA-CS

**Figure 6:**
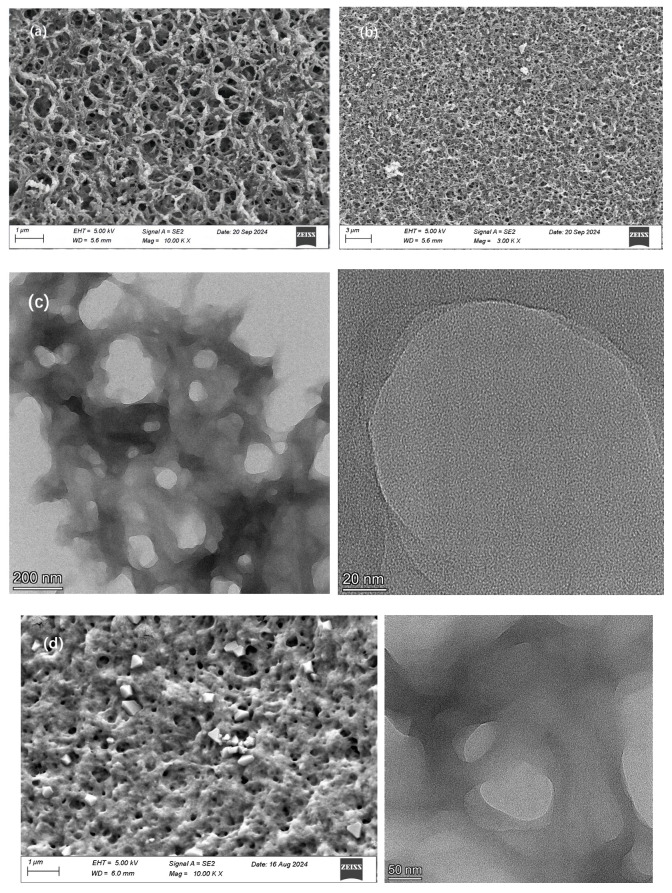
(**a**,**b**) SEM image of PEPA-CS aerogel. (**c**) TEM image of PEPA-CS aerogel. (**d**) SEM and TEM image of pure CS aerogel.

The morphological characteristics of the CS-grafted PEPA aerogel were investigated using scanning electron microscopy (SEM). This is shown in Figure 6. By comparing the SEM images of the pure aerogel and modified aerogel, it can be observed that the surface of the modified aerogel is riddled with numerous pores, resulting in a high porosity and a well-developed porous structure. This extensive specific surface area is conducive to the adsorption of carbon dioxide.

Concurrently, the SEM images depict the porous nature of the product, and the spherical sections reveal that the interior of the aerogel also exhibits a porous structure, a fact that is further substantiated by the TEM images, which elucidate the internal porous framework. The internal powder structure of the chitosan aerogel beads is well-supported, and this structure, which has been crosslinked and modified, is termed a layered porous structure. This robust supportive framework endows the modified chitosan aerogel with more stable strength characteristics. Additionally, the internal porous structure provides a greater specific surface area, which is conducive to the adsorption of CO_2_ [28]. Given that the aerogel beads prepared in the experiment are at the millimeter level, the interior of the product is not the primary adsorption site. The main adsorption site is the surface. The porous structure inside can increase the adsorption capacity of CO_2_ and provide stability for the aerogel structure [29].

### 3.5. N_2_ Absorption Isotherms of the Samples

The specific surface area (SSA) was measured by N_2_ adsorption/desorption isotherms at −196 °C with a Micromeritics ASAP 2020 instrument, using the Brunauer−Emmett−Teller (BET) method [30].

**Figure 7:**
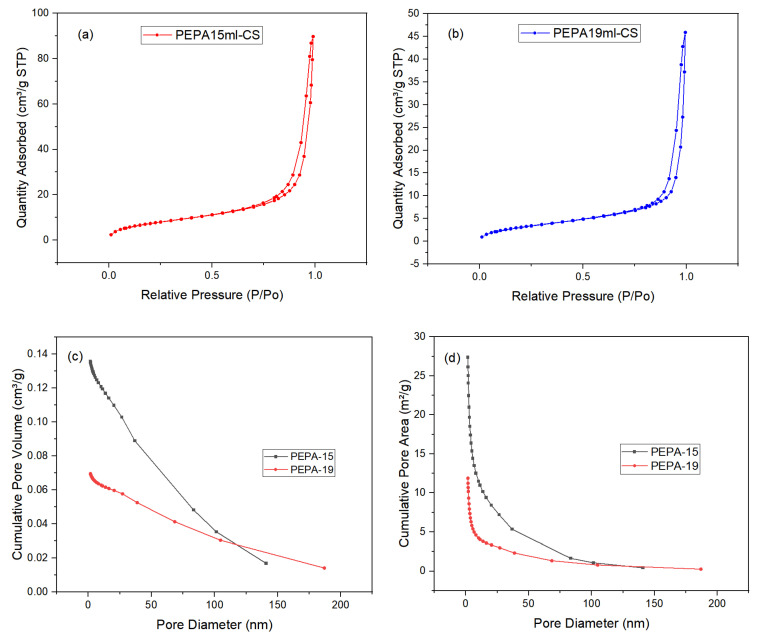
N_2_ adsorption–desorption isotherm of (**a**) PEPA-15, (**b**) PEPA-19, (**c**) Cumulative Pore Volumes, (**d**) Cumulative Pore Area.

To further evaluate the pore structure of the samples, their N_2_ adsorption–desorption isotherms were obtained at 77 K. The results are shown in Figure 7. Figure 7a,b are the N_2_ adsorption–desorption curves of PEPA-15 and PEPA-19, both of which are type IV isotherms of mesoporous materials with hysteresis loop characteristics [31]. The BET model was used to calculate the specific surface area of the sample, and PEPA15 had a larger specific surface area, which was conducive to the adsorption of carbon dioxide [32]. The specific surface area of PEPA15 was 28.4578 m^2^/g, which decreased to 12.2333 m^2^/g with the addition of 19 mL of PEPA, which may be due to pore clogging due to PEPA agglomeration [33,34]. In addition, the surface ammonium carbamate formed after carbon dioxide adsorption forms an electrostatically connected network, which hinders the further diffusion of carbon dioxide in the whole [35]. Figure 7c, d demonstrates that, within the pore size range of 1.0–20.0 nm, there is a rapid increase in the cumulative pore volume and cumulative specific surface area of the material, indicating the presence of a significant number of micropores with sizes between 1.0 and 20.0 nm within the material [36]. The average pore size of PEPA-CS aerogel was 19.5127 nm by BET analysis, and the average pore size of PEPA-CS aerogel was shown in Figure 6c can further demonstrate that the average pore diameter of the product is around 20 nm. The pore structure of carbon materials plays a critical role in CO_2_ capture, and a high specific surface area, large micropore volume, and narrow aperture are benefits for adsorption.

### 3.6. The Surface Chemical Compositions of the Samples (XPS)

**Figure 8:**
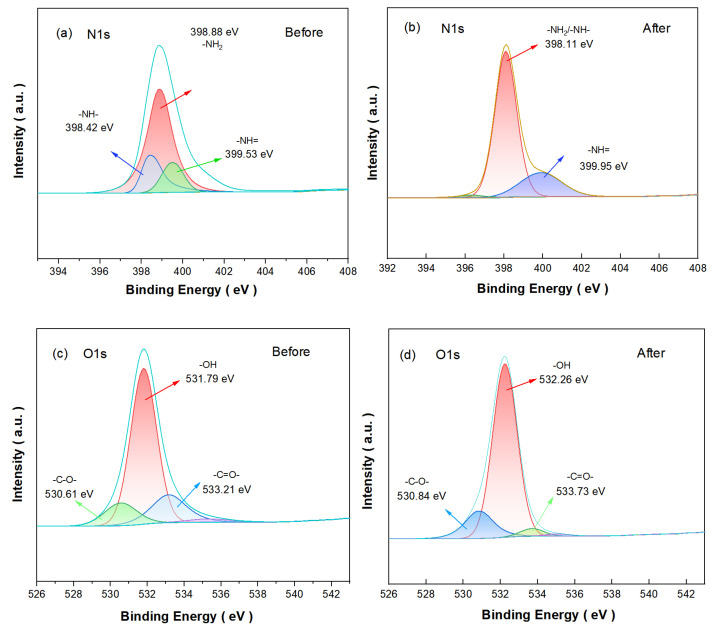
XPS spectra of (**a**,**b**) N 1s and (**c**,**d**) O 1s before and after modification.

In order to determine the PEPA-CS binding mechanism, XPS was used to compare the changes in aerogels before and after modification. In the XPS spectra of the CS coagulants after PEPA loading, the high-resolution spectra of N 1s before and after PEPA-CS modification (Figure 7a,b) showed that the characteristic peak corresponding to -NH_2_/-NH- changed from 398.88 eV to 398.11 eV, indicating that the density of the electron cloud around the N atom increased. The corresponding characteristic peak of -N= changed from 399.53 eV to 399.95 eV, and the displacement did not change much, which proved that it did not participate in the modification. In the high-resolution spectra of O 1s of PEPA-CS, the characteristic peaks at 533.73 eV, 532.26 eV, and 530.83 eV belong to -C=O-, -OH, and -C-O-functional groups, respectively (Figure 8d) [34]. After the modification, the peaks of -C=O- and -OH were shifted from 533.21 eV and 531.79 eV to 533.73 eV and 532.26 eV, which was the result of grafting, which proved that there was chemical grafting between amino and hydroxyl functional groups.

In summary, it is the reaction of the amino group and the hydroxyl group that realizes the grafting of the amino group to the surface of the chitosan aerogel.

### 3.7. CO_2_ Adsorption Capacity

It is known that the adsorption mechanism of the amino-modified adsorbent adsorbing carbon dioxide proceeds through the following three pathways [37].

$${CO}_{2}+{2RNH}_{2}\;\to \;{RNCOO}^{-}+{RNH}_{3}^{+}$$

$${CO}_{2}+{2R}_{2}NH\;\to \;R_{2}{NCOO}^{-}+R_{2}{NH}_{2}^{+}$$

$${CO}_{2}+R_{2}{NH}_{2}+R^{\prime }{NH}_{2}\;\to \;R_{2}{NCOO}^{-}+R^{\prime }{NH}_{3}^{+}$$

In order to determine the optimal conditions for distributing as many amino groups as possible on the aerogel surface, we studied in detail the effect of concentrated modifier concentration on the amount of carbon dioxide adsorbed.

With regard to amine modified materials, it is well-known that the amine content is an important factor influencing the CO_2_ adsorption capacity [38,39,40,41,42]. In this experiment, the optimal conditions for the best carbon dioxide adsorption effect were explored by changing the amount of amino modifier.

The optimal conditions for the preparation of aerogel were PEPA-15 mL; the reaction time was 6 h and the reaction temperature was 60 °C. By comparing the adsorption effect of pure aerogels, the adsorption capacity of pure aerogels was 0.12 mmol/g at 25 °C and 1 bar, and the carbon dioxide adsorption capacity of CS aerogels modified by PEPA increased from 0.89 mmol/L to 1.59 mmol/L. Comparing the BET and adsorption test results of the pure CS aerogel and modified aerogel, the adsorption capacity of the modified chitosan aerogel is much greater than that of the pure chitosan aerogel, and if the concentration of the modifier is too large or too small this will also lead to a reduction in carbon dioxide adsorption. So, it is concluded that the adsorption of CO_2_ is achieved by the combination of amino chemisorption [43], surface van der Waals action, and physical adsorption of electrostatic interactions.

**Figure 9:**
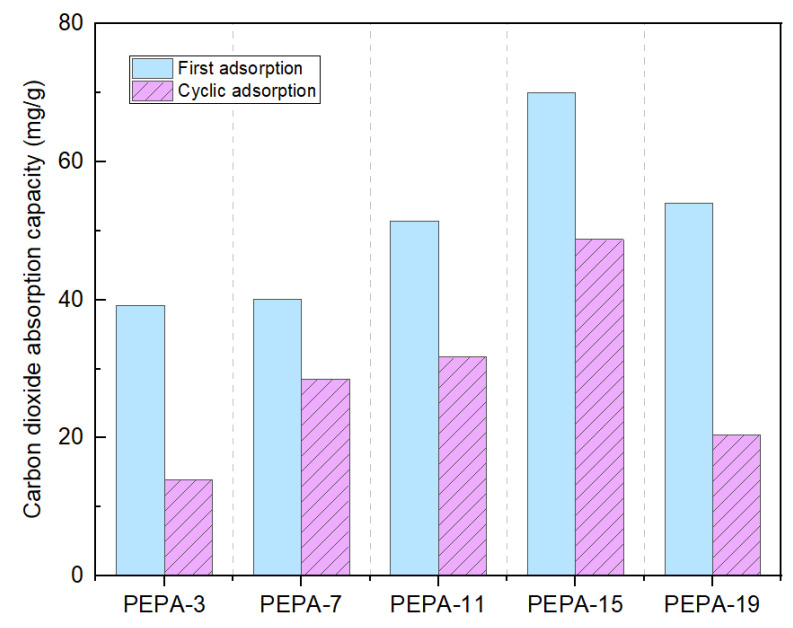
CO_2_ adoption of PEPA-3, PEPA-7, PEPA-11, PEPA-15, PEPA-19.

Due to the magneton stirring in the carbon dioxide absorption kettle, as shown in Figure 9, the spherical structure of the aerogel was broken after the first adsorption, resulting in an overall reduction of about 20 mg/g in the secondary adsorption process. Despite the increase in N content, the CO_2_ adsorption of 19 mL of the PEPA-modified CS aerogel was lower than that of 15 mL the of PEPA-modified CS aerogel due to the relatively greater effect of the reduced specific surface area.

**Figure 10:**
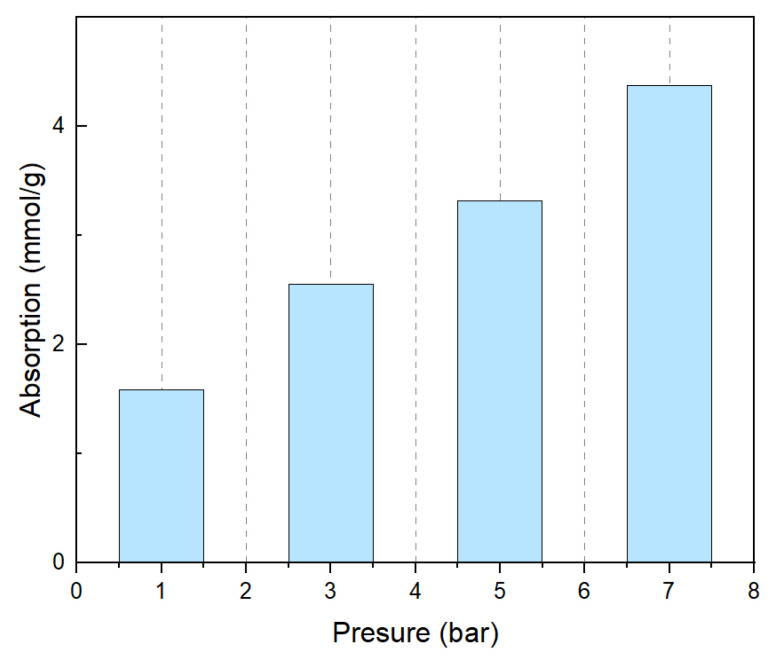
CO_2_ absorption capacity of PEPA-15 at different pressures.

At the same time, we also tested the CO_2_ absorption capacity of PEPA-15 at different pressures, as shown in Figure 10, and the adsorption capacity gradually increased at 1 bar, 3 bar, 5 bar, and 7 bar pressures. It has been observed that, as the pressure increases, the adsorption capacity of CO_2_ also gradually rises, indicating that pressure is a significant factor influencing the adsorption performance of CO_2_ solid adsorbents. One study [44] developed a porous liquid based on flexible covalent organic frameworks (COFs), known as COF-PLs, which significantly enhanced CO_2_ adsorption and catalytic efficiency through a dynamic expansion effect. The research revealed that the pores of COF-PLs dynamically adjust in response to changes in CO_2_ pressure. This dynamic expansion effect not only provides additional gas adsorption capacity (reaching 7.04 mmol/g at 40 bar) but also facilitates the mass transfer of gas molecules during the catalytic process. By optimizing the preparation conditions of the adsorbent and operational parameters, the adsorption efficiency of the adsorbent under different pressures can be significantly improved. Another study [29] optimized the preparation conditions of an amine-modified silica aerogel (AMSA) using response surface methodology (RSM) to enhance its adsorption capacity and adsorption–desorption stability under low-pressure CO_2_ conditions. The study found that, as the partial pressure of CO_2_ increases, the adsorption capacity also increases. The adsorption equilibrium and kinetics under different CO_2_ partial pressures and temperatures were investigated by fitting with the Freundlich isotherm model and the bi-exponential model. By comparing the aforementioned studies and the results obtained, it can be concluded that optimizing adsorption conditions and operational parameters can significantly enhance the adsorption efficiency of adsorbents under various pressures.

## 4. Conclusions

In this study, chitosan aerogels for carbon dioxide adsorption were prepared via grafting, and the successful grafting of PEPA onto the chitosan (CS) surface was confirmed by X-ray photoelectron spectroscopy (XPS) and Fourier-transform infrared spectroscopy (FTIR). The experimental results indicated that the adsorption capacity of pure chitosan aerogels was significantly inferior to that of the modified chitosan aerogels. Meanwhile, the BET analysis of PEPA-15 and PEPA-19 revealed that the CO_2_ adsorption capacity of the polymer aerogel is not only dependent on the available amine loading on the polymer surface but also closely related to the actual porosity of the product. Grafting a higher concentration of PEPA resulted in a decreased adsorption capacity and inferior physical properties. When the optimal conditions were selected, the maximum CO_2_ adsorption capacity reached 1.59 mmol/g. Moreover, measurements of the circulating CO_2_ capacity indicated that the regeneration process could be easily achieved by heating the sample to 90 °C, which facilitated the reuse of the aerogel and reduced the desorption cost.

These results suggest that the prepared CO_2_-trapping polymer aerogels hold significant potential for industrial applications. Future research could further explore other modification methods, such as impregnation or encapsulation, to optimize the performance of the aerogels. Additionally, expanding the specific surface area of the product and enhancing its adsorption performance through physical or chemical methods could further explore the optimal conditions for the adsorption of carbon dioxide by PEPA-modified chitosan aerogels. This would not only help improve the adsorption efficiency of the aerogel but also provide more economical and sustainable solutions for industrial applications.

## Data Availability

Raw data can be made available freely upon request from the corresponding authors.
